# The efficacy and safety of chemoimmunotherapy in patients with MSI-L/MSS/pMMR status metastatic colorectal cancer: a systematic review and meta-analysis of randomized controlled trials

**DOI:** 10.3389/fonc.2025.1514485

**Published:** 2025-06-12

**Authors:** Qi-Jing Zhang, Jia-xin Zhou, Da-hai Hu, Jing-hua Pan, Si-min Luo, Qi Yao

**Affiliations:** ^1^ Department of General Surgery, The First Affiliated Hospital of Jinan University, Guangzhou, China; ^2^ School of Medicine, Jinan University, Guangzhou, Guangdong, China; ^3^ International School, Jinan University, Guangzhou, Guangdong, China; ^4^ Department of Sports Medicine, The First Affiliated Hospital, Guangdong Provincial Key Laboratory of Speed Capability, The Guangzhou Key Laboratory of Precision Orthopedics and Regenerative Medicine, Jinan University, Guangzhou, China; ^5^ Chaoshan Hospital, The First Affiliated Hospital of Jinan University, Chaozhou, China; ^6^ Department of Bone and Joint Surgery, The First Affiliated Hospital, Jinan University, Guangzhou, China; ^7^ Department of Anorectal Surgery, The Second Clinical Medical College, Jinan University (Shenzhen People’s Hospital), Shenzhen, China

**Keywords:** colorectal cancer, immune checkpoint inhibitors, efficacy, safety, meta-analysis

## Abstract

**Background:**

Colorectal cancer (CRC) remains a leading cause of cancer-related mortality worldwide, necessitating innovative therapeutic approaches. Most patients with CRC exhibit microsatellite instability-low/stable (MSI-L/MSS) or proficient mismatch repair (pMMR) status, with chemotherapy being the standard first-line treatment. Chemoimmunotherapy, incorporating immune checkpoint inhibitors (ICIs), has emerged as a potential treatment for MSI-L/MSS/pMMR CRC. This study aimed to comprehensively evaluate the efficacy and safety of chemoimmunotherapy in metastatic CRC (mCRC) patients with MSI-L/MSS/pMMR status.

**Methods:**

A systematic search of PubMed, EMBASE, ScienceDirect, and Cochrane Library was conducted in accordance with PRISMA guidelines, targeting studies published between May 2022 and September 2024. The meta-analyses utilized the generic inverse-variance method with a random effects model.

**Results:**

Four studies encompassing 934 patients with mCRC met the inclusion criteria. The meta-analysis revealed a significant reduction in the risk of progression or death with chemoimmunotherapy compared with chemotherapy (HR: 0.82, 95% CI: 0.70–0.97, *P* = 0.02). Subgroup analyses based on sex (male vs. female) and ECOG status consistently demonstrated a significant benefit of chemoimmunotherapy in MSI-L/MSS/pMMR tumors. Adverse event analysis indicated an increase in adverse events in the chemoimmunotherapy group.

**Conclusion:**

Existing evidence indicates a statistically significant and clinically meaningful benefit in PFS with chemoimmunotherapy, albeit with a slight increase in all-grade and high-grade toxicities compared to chemotherapy. Future research focusing on biomarkers and innovative treatments is essential for enhancing patient outcomes.

**Systematic review registration:**

https://www.crd.york.ac.uk/PROSPERO/, identifier CRD42024520150.

## 1 Introduction

Colorectal cancer (CRC) has long been one of the top three most prevalent cancer types worldwide, with 1.9 million new cases and 900,000 deaths reported in 2022, making it the second leading cause of cancer-related mortality, responsible for nearly one in ten cases and deaths (1). In the United States, it was estimated that 117,550 males and 71,160 females would be diagnosed with CRC, while 28,470 males and 24,080 females would succumb to this disease in 2023 (2). Multiple studies have indicated a rising incidence of CRC among individuals under 50 years old in various high-income countries (3–7). This trend is strongly linked to factors such as alcohol consumption, smoking, consumption of red or processed meat, body fat, and antibiotic use affecting the gut microbiome (8, 9).

Outcomes in patients with advanced CRC remain poor, underscoring the urgent need for new treatment modalities. Based on the DNA mismatch repair (MMR) system, CRC can be classified into microsatellite instability-high/deficient mismatch repair (MSI-H/dMMR), microsatellite instability-low/stable (MSI-L/MSS) and proficient mismatch repair (pMMR) status. Immunotherapy has shown efficacy in tumors with dMMR/MSI-H (10). It is widely acknowledged that immune checkpoint inhibitors (ICIs) have revolutionized the treatment of patients with metastatic CRC (mCRC) with dMMR/MSI-H status. The National Comprehensive Cancer Network (NCCN) guidelines recommend Pembrolizumab, Nivolumab, and Ipilimumab for dMMR/MSI-H disease in the first-line setting (11).

However, ICIs therapy for mCRC has several limitations. Only about 4–5% of colorectal tumors exhibit dMMR or MSI-H, while the vast majority of MSI-L/MSS/pMMR tumors are insensitive to immunotherapy (12).The combination of fluorouracil (plus leucovorin) and either irinotecan (FOLFIRI) or oxaliplatin (FOLFOX) plus bevacizumab remains the standard first-line treatment for mCRC (13–15). Disappointing results have been observed with immunotherapy in patients with MSI-L/MSS/pMMR CRC (16). Evidence suggests that patients with MSI-L/MSS/pMMR CRC hardly benefit from monotherapy with ICIs. Previous studies, such as the KEYNOTE-016 and KEYNOTE-028 trials, included 18 and 22 patients with MSS mCRC, respectively, who were treated solely with pembrolizumab, and observed no response to immunotherapy (16, 17). Furthermore, the IMblaze370 trial demonstrated that MSS/pMMR CRC patients did not benefit from monotherapy with ICIs in progression-free survival (PFS) and overall survival (OS) when compared to a targeted drug (18).

Given the poor outcomes in patients with advanced CRC, the exploration of new treatments is critical. Future directions for treating MSI-L/MSS/pMMR CRC may involve combining drug therapies, such as chemoimmunotherapy, to improve patient outcomes. Nonetheless, the role of chemoimmunotherapy in the treatment of MSI-L/MSS/pMMR type mCRC remains uncertain. The efficacy of adding ICIs to the standard chemotherapy regimen for MSI-L/MSS/pMMR CRC compared with conventional chemotherapy has not been conclusively established. Therefore, it is imperative to conduct a meta-analysis of various clinical trials involving MSI-L/MSS/pMMR type mCRC to evaluate the safety and efficacy of ICIs.

## 2 Materials and methods

### 2.1 Literature search

This meta-analysis was registered in the PROSPERO database (No. CRD42024520150). The systematic review was conducted according to the Preferred Reporting Items for Systematic Reviews and Meta-Analysis (PRISMA) guidelines (19). We retrieved the PubMed, EMBASE, ScienceDirect, and Cochrane Library databases to systematically filter published studies from December 2020 to April 2024 for present research. The search terms and keywords were “metastatic” OR “advanced” AND “colorectal neoplasm” OR “colorectal tumor” AND “immunotherapy” OR “immune checkpoint inhibitor” OR “nivolumab” OR “ipilimumab” OR “sintilimab” OR “durvalumab” OR “atezolizumab” OR “pembrolizumab” OR “avelumab” OR “camrelizumab” OR “tislelizumab” OR “tremelimumab” OR “toripalimab”. All entries that met these criteria were manually searched for.

### 2.2 Inclusion and exclusion criteria

The inclusion criteria were as follows: (1) randomized clinical trials (RCTs) that compared the efficacy and safety of ICIs in combination with chemotherapy, against chemotherapy alone for the treatment of mCRC; (2) studies published in English; (3) available hazard ratios (HRs) for progression-free survival (PFS), overall survival (OS), or a clear Kaplan-Meier curve. The exclusion criteria were as follows: (1) reviews, perspectives, observational studies, case reports, and study protocols; (2) single-arm clinical trials; (3) the use of ICIs in both arms; (4) studies with limitations on MSI-H/dMMR CRC (5) repetitive publications of one study; (6) studies with unbalanced matching procedures, or incomplete data.

### 2.3 Data extraction

**Figure 1:**
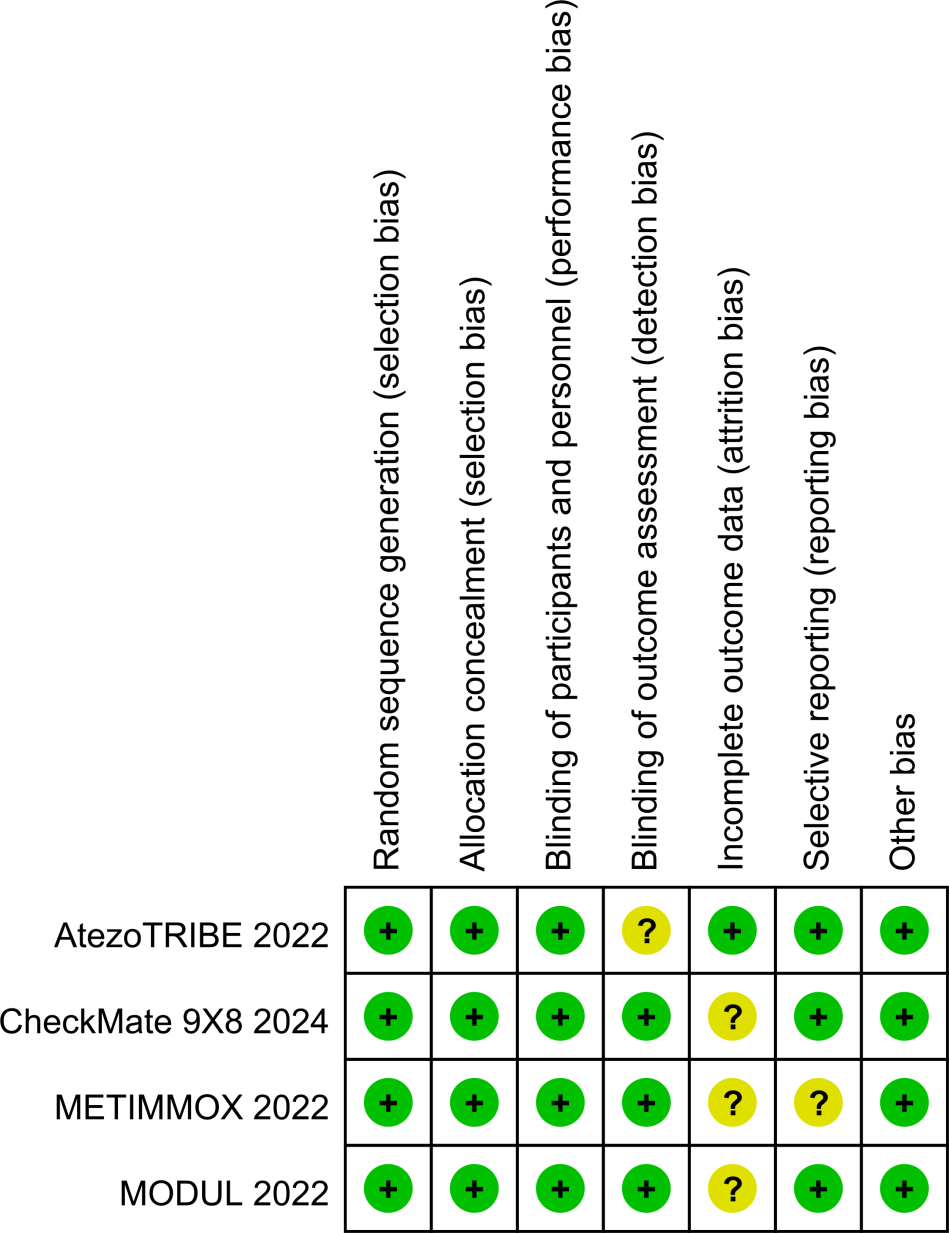
Risk of bias summary of RCTs. +low risk,? unclear risk,−high risk.

**Figure 2:**
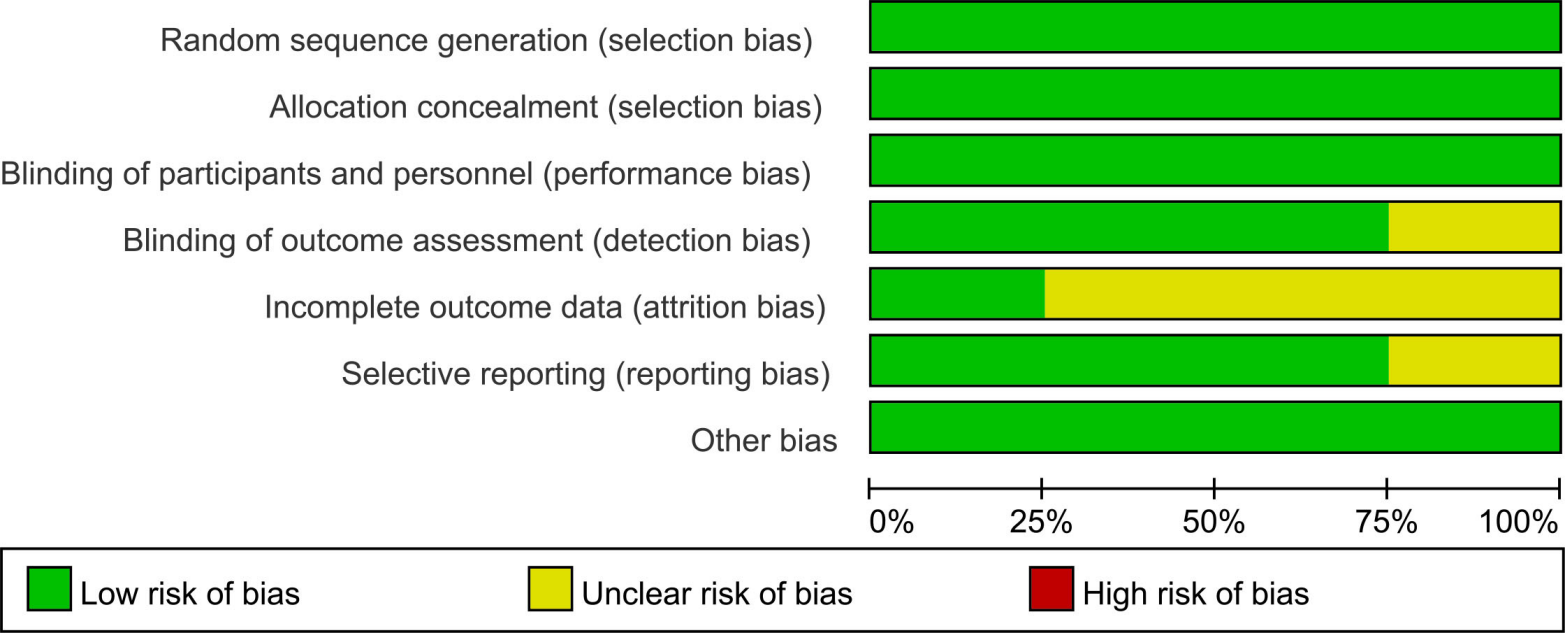
Risk of bias assessment of RCTs using the weighted summary.

Two authors (QJZ and JXZ) independently extracted data. Any disagreement was resolved by discussion until consensus was reached or by consulting a third author (DHH). The following data were extracted: author, year of publication, country where the study was conducted, clinical trial phase, total number of individuals included in the study, microsatellite status, remedies of the experimental and control arms, HRs for PFS and OS, stable disease, disease control, all-grade and high-grade (grade 3 or higher) adverse events, fatal adverse events, median follow-up, etc. Multiple reports from a single study were treated as a single data point, prioritizing the results based on the most complete and appropriately analyzed data. We also extracted the HRs using the Engauge Digitizer 11.1 and Excel. When critical data were missing or unclear in the published reports, supplemental data were requested from the study authors. The risk of bias in the included studies was independently assessed by two authors (QJZ and JXZ) using the Risk of Bias Tool (version 2), which evaluates the following domains: Random sequence generation (selection bias), Allocation concealment (selection bias), Blinding of participants and personnel (performance bias), Blinding of outcome assessment (detection bias), Incomplete outcome data (attrition bias), Selective reporting (reporting bias), Other potential sources of bias (e.g., baseline imbalance). Any discrepancies were resolved through discussion with the senior author (DHH). The results of the risk of bias assessment are presented in **Figures 1**, **2**. Each study was categorized as having a low, unclear, or high risk of bias for each domain, and an overall risk of bias assessment was made.

### 2.4 Statistical analysis

The meta-analysis was conducted using the generic inverse-variance method accompanied by a random-effects model to account for potential variations across studies. The primary objective was to compare the PFS between the experimental and control groups. Subgroup analyses for PFS were also performed based on sex and ECOG performance status. Secondary objectives included comparing OS, and all-grade and high-grade (grade 3 or higher) adverse events between the two groups. The principal summary measures were HRs with 95% two-sided confidence intervals (CIs) for PFS and OS, and odds ratios (ORs) with 95% two-sided CIs for adverse events. All statistical analyses were performed using Review Manager software, version 5.4 (The Nordic Cochrane Center, The Cochrane Collaboration, Copenhagen, Denmark). Heterogeneity within each subgroup was assessed using Higgins’ I-squared (I²) statistics to determine the degree of variation attributable to heterogeneity rather than chance. A *P*-value of less than 0.05 was considered statistically significant.

## 3 Results

### 3.1 Study selection

**Figure 3:**
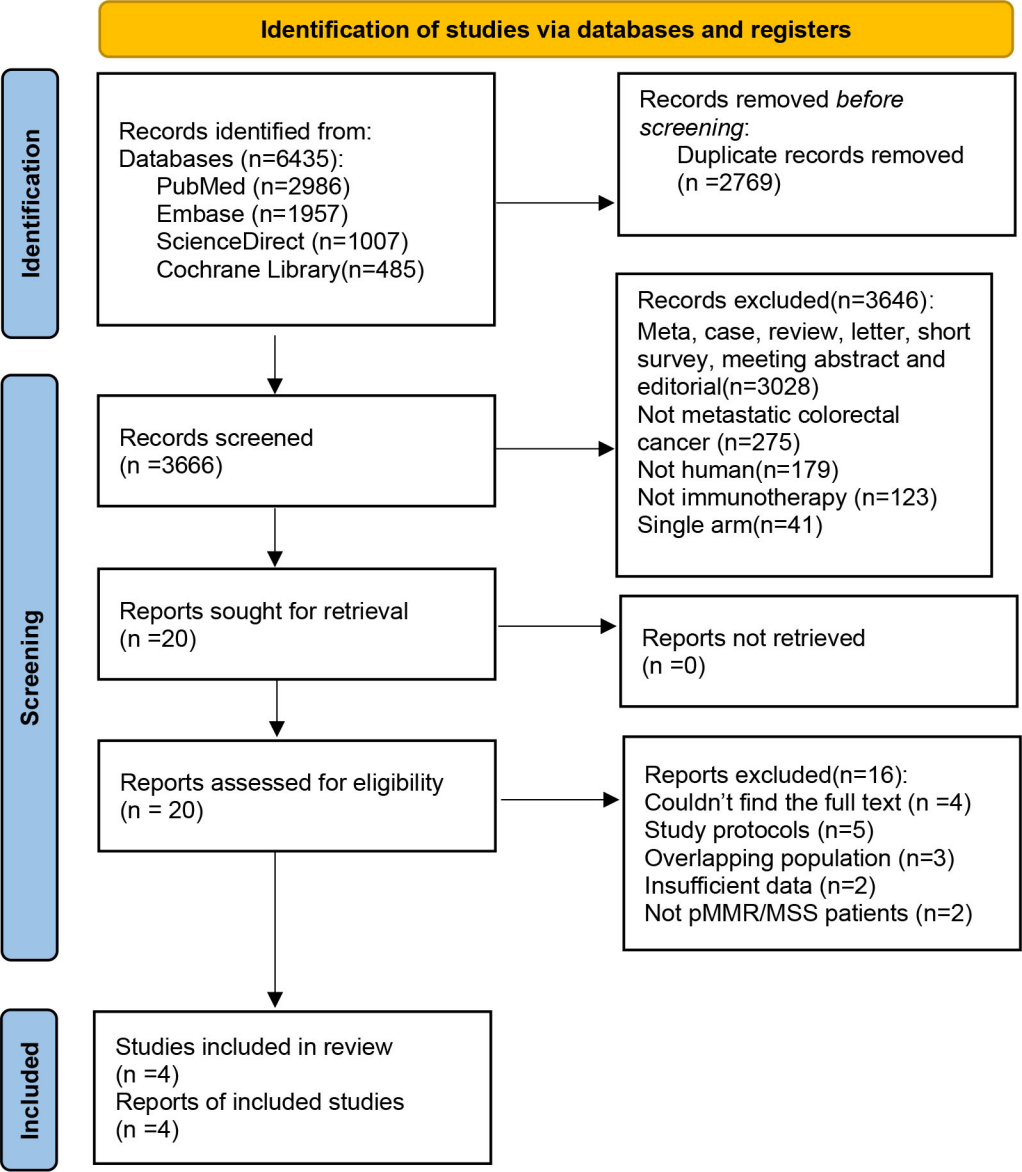
PRISMA flow chart showing the selection of articles for systematic review and meta-analysis.

The initial literature search identified a total of 6,435 reports, including 2,986 reports from PubMed, 1,957 reports from EMBASE, 1,007 reports from ScienceDirect, and 485 reports from the Cochrane Library. After removing duplicate entries, 3,666 reports were deemed eligible. Following a detailed evaluation based on predefined inclusion and exclusion criteria, a final selection of four studies was conducted. The flowchart of study selection is presented in **Figure 3**.

### 3.2 Baseline characteristics of included studies

The characteristics of all the included studies are shown in **Table 1**. A total of 934 patients were included in the four studies, with publication years ranging from 2022 to 2024. All studies were RCTs. In the experimental arms, all studies combined an ICI with chemotherapy, whereas the control arms used standard chemotherapy. In these studies, the vast majority or all patients had MSI-L/MSS/pMMR status. The median follow-up was reported in three studies, ranging from 20.3 months to 45.2 months.

**Table 1:** Characteristics of included studies.

Trial name, year	Country	Phase	Number of patients	Gender (M/F)	MSS/MSI/ missing date	Experimental arm (n)	Control arm (n)	OS (median or %)	PFS/DFS/EFS (median or %)	Stable disease (SD) (%)	Disease control (%)	Grade ≥ 3 TRAEs, (%)	Fatal AEs, no.	Median follow-up (Months)
AtezoTRIBE, 2022 (48, 49)	Italy	II	218	125/93	202/13/3	FOLFOXIRI + bevacizumab +atezolizumab (145)	FOLFOXIRI+ bevacizumab (73)	33.0 vs. 27.2 Months (median)	13.1 vs. 11.5 Months (median)	NA	NA	67 vs. 61	2 vs. 0	45.2
METIMMO, 2022 (50)	Norway	II	76	41/35	76/0/0	FLOX+ nivolumab (38)	FLOX only (38)	NA	8.8 vs. 10.0 Months (median)	NA	NA	NA	NA	NA
MODUL cohort 2, 2022 (51)	Multi-national	II	445	271/ 174	371/7/67	Fluoropyrimidine+ bevacizumab +atezolizumab (297)	Fluoropyrimidine+ bevacizumab (148)	NA	7.1 vs. 7.4 Months (median)	NA	76.4 vs. 75.0	37.5 vs. 30.1	3 vs. 1	20.3
CheckMate 9X8, 2024 (52)	Multi-national	II	195	119/76	182/13/0	Nivolumab + SOC (mFOLFOX6/bevacizumab) (127)	SOC (68)	29.2 vs. NR Months (median)	11.9 vs. 11.9 Months (median)	31 vs. 38	NA	75 vs. 48	2 vs. 2	23.7 vs. 23.2

### 3.3 The comparison of PFS and OS with chemoimmunotherapy versus standard chemotherapy

**Figure 4:**
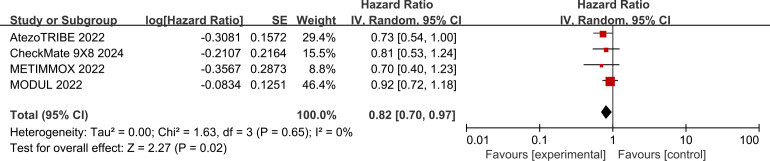
Forest plots for PFS between research and control groups in mCRC patients.

In the pooled analysis of four studies, the use of ICIs was associated with a 18% reduction in the risk of progression or death compared with standard chemotherapy (HR: 0.82, 95% CI: 0.70–0.97, *P* = 0.02) (**Figure 4**). The included studies exhibited a low degree of heterogeneity (I² = 0%, *P* = 0.65).

**Figure 5:**
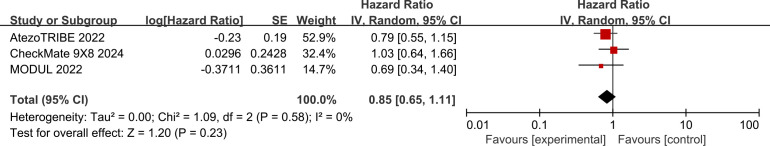
Forest plots for OS between research and control groups in mCRC patients.

Data regarding OS were available from three studies. The use of ICIs was associated with a relatively lower risk of death than chemotherapy (HR: 0.85, 95% CI: 0.65–1.11) (**Figure 5**). The included studies had a low degree of heterogeneity (I² = 0%, *P* = 0.58). However, the difference between the two groups was not statistically significant (*P* = 0.23).

### 3.4 Subgroup analyses for PFS

**Figure 6:**
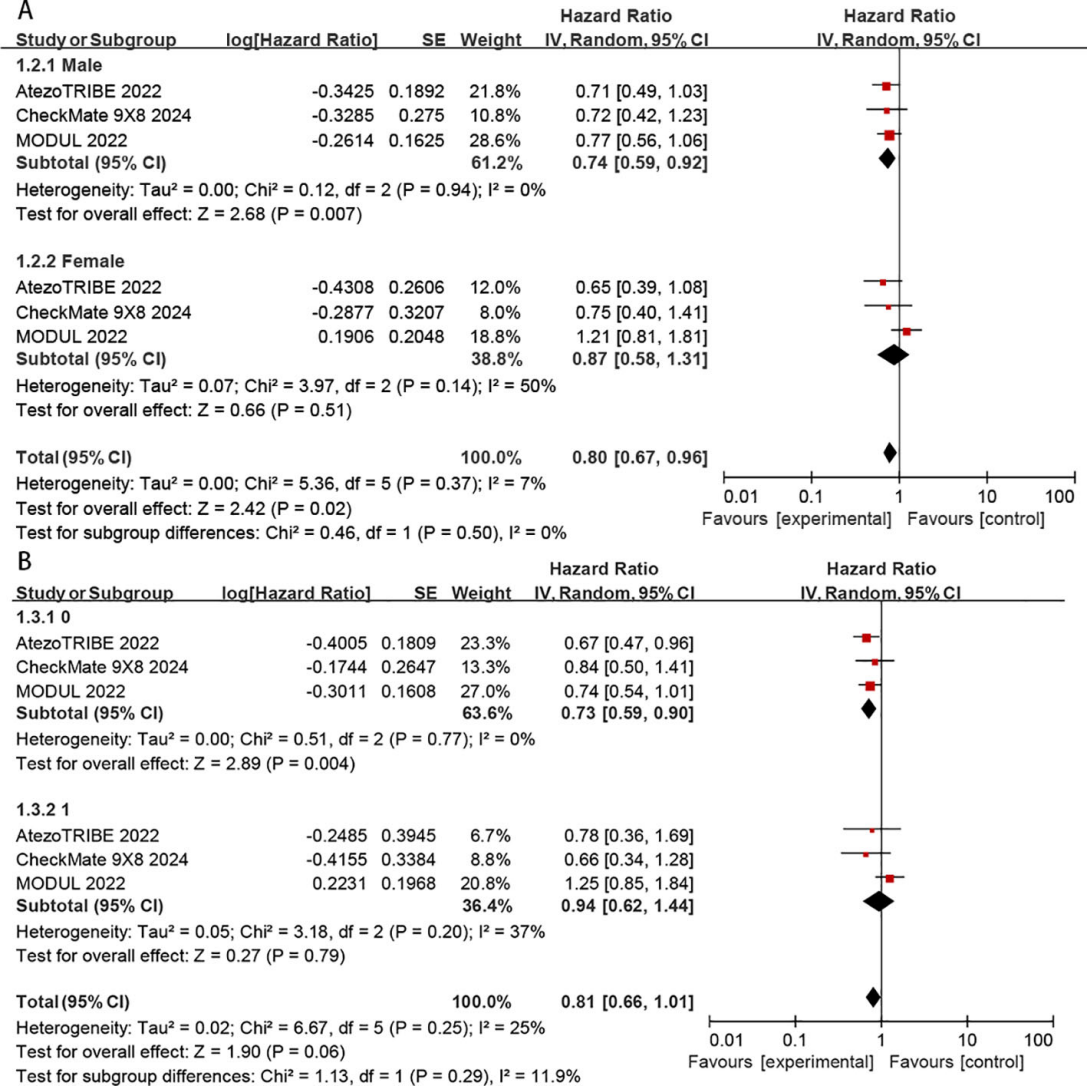
Subgroup analyses according to sex **(A)** and ECOG status **(B)**.

Subgroup analyses of PFS were conducted according to sex (male vs. female) and ECOG status (0 vs.1). The benefit of chemoimmunotherapy was consistent across the evaluated subgroups without any apparent differences (*P* > 0.05 for test of subgroup differences) (**Figures 6A, B**). However, the benefit of immunotherapy was significantly greater in patients with ECOG = 0 (HR: 0.73, 95% CI: 0.59–0.90, *P* = 0.004) than in those with ECOG = 1 (HR: 0.94, 95% CI: 0.62–1.44, *P* = 0.79) (**Figure 6B**).

### 3.5 The comparison of adverse events with chemoimmunotherapy versus standard chemotherapy

**Figure 7:**
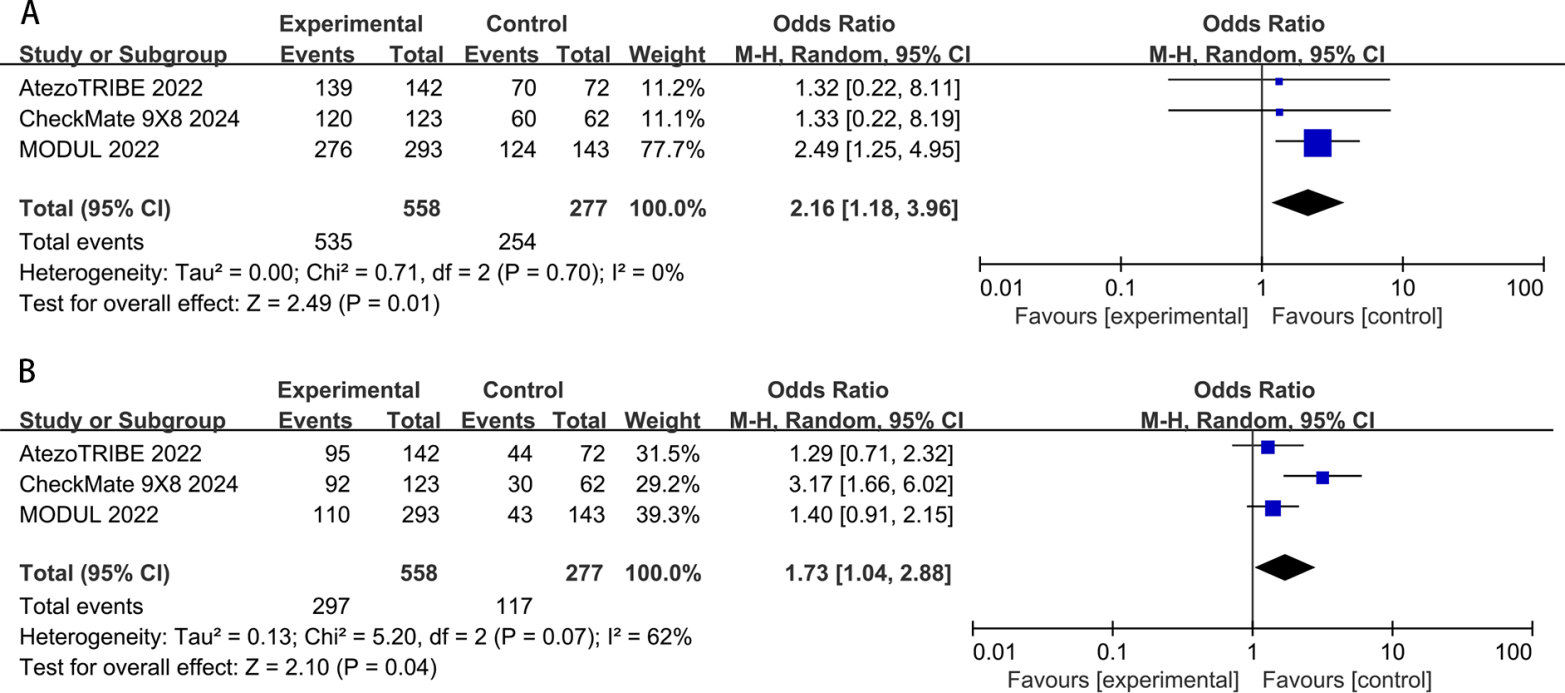
All-grade **(A)** and high-grade **(B)** adverse events rates for research and control arm.

In the pooled data of the three studies, all-grade adverse events in the ICIs groups (95.9%) were slightly higher than those in the standard chemotherapy groups (91.7%). The difference was of conspicuous significance (OR: 2.16, 95% CI: 1.18–3.96, *P* = 0.01). The result showed low heterogeneity (*P*=0.70, I^2^ = 0%) for all-grade adverse events (**Figure 7A**). The incidence of high-grade (grade 3 or higher) adverse events in the chemoimmunotherapy group (53.2%) was slightly higher than that in the chemotherapy group (42.2%). A significant difference was observed between the two groups (OR:1.73, 95% CI: 1.04–2.88, *P*=0.04). Moderate heterogeneity was observed in these studies (*P*=0.07; I^2^ = 62%) (**Figure 7B**).

## 4 Discussion

In the present meta-analysis of four RCTs comparing chemotherapy, we observed a modest improvement in PFS with chemoimmunotherapy (HR = 0.82). This minor benefit, however, was accompanied by a slight increase in both all-grade and high-grade toxicities. The relatively small gain in PFS must be weighed carefully against the associated risks, as these adverse events may affect the overall benefit–risk balance. The clinical significance of such modest improvements in PFS should be critically evaluated in the context of higher toxicity. While chemoimmunotherapy showed consistent benefits across all evaluated subgroups, immunotherapy demonstrated significantly greater efficacy in patients with better performance status (ECOG = 0) compared to those with poorer status (ECOG = 1). Further clinical studies are warranted to determine whether these marginal benefits justify the significantly increased risk of adverse events. To the best of our knowledge, our meta-analysis is the most current in this domain, incorporating the latest Checkmate 9X8 study in its analysis.

The failure to observe a statistically significant improvement in OS despite the benefit seen in PFS is a critical finding in this analysis. There are several potential reasons why improvements in PFS may not necessarily translate into OS advantages, particularly in the context of chemoimmunotherapy. First, immunotherapy, especially ICIs, can have delayed effects on OS. It is well-documented that ICIs can lead to long-term remissions in a subset of patients, even after the disease initially progresses. The long-term outcomes of patients responding to anti-PD-1/PD-L1 therapy have been explored in a study that analyzed data from a phase I trial. The findings revealed that patients who responded to these therapies experienced significant OS benefits, with some achieving complete responses and no deaths occurring during the follow-up period. This underscores the potential of ICIs to induce durable remissions and highlights the importance of evaluating patient stratification strategies to optimize treatment outcomes (20).These delayed survival benefits may not be captured within the timeframe of the studies included in this analysis, which could have influenced the failure to observe a statistically significant OS benefit. Second, the lack of significant OS benefit could also be attributed to immune-related adverse events, which may affect survival independently of the cancer itself. In this analysis, the higher rate of high-grade adverse events observed in the chemoimmunotherapy group could potentially contribute to treatment-related mortality, which might obscure the survival benefit of chemoimmunotherapy. Last, Larger trials with longer follow-up periods could provide more definitive data on the OS impact of chemoimmunotherapy.

All four studies utilized combinations such as oxaliplatin and 5-fluorouracil (5-FU) with ICIs. According to preclinical data, combining a PD-1/PD-L1 inhibitor with an immunogenic cell death inducer like oxaliplatin could enhance the efficacy of immunotherapy (21–23). Moreover, 5-FU can eliminate myeloid-derived suppressor cells and mitigate tumor-induced immunosuppression (24, 25). Therefore, the combination of 5-FU and oxaliplatin could potentially improve the anti-tumor immune response.

Previous studies have demonstrated that ICIs provide unique response and survival benefits compared with chemotherapy in patients with advanced dMMR/MSI-H CRC. Immunotherapy can achieve a significantly longer PFS with fewer treatment-related adverse events. The KEYNOTE-177 study has successfully reshaped the guidelines, making pembrolizumab the standard of care for first-line treatment of MSI-H mCRC. Currently, there are no treatment guidelines specifying the use of ICIs for MSI-L/MSS/pMMR mCRC. However, our results suggest that chemoimmunotherapy, compared with chemotherapy, can improve PFS and OS in these patients. It is important to emphasize that the observed benefit of chemoimmunotherapy in our meta-analysis was significantly lower in patients with MSI-L/MSS/pMMR tumors (HR: 0.81, 95% CI: 0.68-0.96) compared with MSI-H/dMMR tumors (HR: 0.60, 95% CI: 0.45-0.80) according to KEYNOTE-177 (10). Researchers believe that the lower benefit of ICIs in patients with MSI-L/MSS/pMMR CRC is due to their resistance to immunotherapy. Compared with MSI-H/dMMR CRC, MSI-L/MSS/pMMR tumors exhibit characteristics such as low tumor mutational burden (TMB), low expression of major histocompatibility complex (MHC) molecules by antigen-presenting cells, inhibited initial activation of T cells, abnormal vascular signaling, and a suppressive tumor immune microenvironment (26). These characteristics collectively suggest that adopting combination therapies to overcome resistance at different stages may be a future direction for treating MSI-L/MSS/pMMR CRC.

Identifying potential beneficiary subgroups within the MSI-L/MSS/pMMR population is an urgent issue, and further research on biomarkers is necessary to customize immunotherapy with checkpoint inhibitors. POLE/POLD1 mutations can serve as potential biomarkers for immunotherapy in MSI-L/MSS/pMMR CRC. Among MSI-L/MSS/pMMR patients receiving ICI treatment, those with POLE/POLD1 mutations had significantly better OS than non-carriers (27–29). Research indicates that POLE and POLD1 mutations are linked to a higher TMB, which can potentially enhance the tumor’s visibility to the immune system, thereby improving the response to ICIs (30, 31) (32). A study focusing on the Chinese population found that patients with POLE/POLD1 damaging variants exhibited significantly higher TMB and a higher frequency of MMR gene variants compared to those without these mutations. This suggested that these genetic alterations could be used to identify patients who might benefit more from immunotherapy (33). As a result, MSI-L/MSS/pMMR patients with these mutations may experience better outcomes with ICI therapy compared to those without such mutations. Another study investigated the use of immune-sensitizing treatment strategies in MSS, O6-Methylguanine-DNA Methyltransferase-Silenced (MGMT) mCRC. The results showed that in MSS, MGMT-silenced mCRC patients, employing temozolomide as initial treatment followed by a regimen combining low-dose ipilimumab and nivolumab holds promise for achieving durable clinical benefits (34). Immunoscore is also undergoing extensive evaluation in multiple patient cohorts and is considered a robust prognostic biomarker. It is a valuable tool in assessing the immune cell infiltration within the tumor microenvironment (TME) of MSI-L/MSS/pMMR CRC. This quantification provides critical insights into the tumor’s immunogenicity, which is essential for understanding the tumor’s potential response to immunotherapy. The immunoscore system evaluates the density and location of immune cells, particularly T lymphocytes, within the tumor and its invasive margin, offering a prognostic indicator that is independent of traditional TNM staging. This system has been integrated into the international WHO classification of Digestive System Tumors, underscoring its clinical utility in patient management and prognosis prediction (35). Recent advancements in understanding the tumor microenvironment have highlighted the role of the immunoscore in guiding treatment decisions. By assessing the density and location of immune cells such as CD8+ T cells within the tumor, the immunoscore provides a comprehensive picture of the immune landscape. This information can be pivotal in designing combination therapies that enhance the efficacy of ICIs in MSI-L/MSS/pMMR CRC (36, 37). In addition to CD3+ and CD8+ T cells, other immune cells within the tumor microenvironment, such as Th17 and memory T cells, have garnered interest in recent years (38). The bottleneck in treating MSI-L/MSS/pMMR CRC lies in transforming the “cold” tumors of MSS into the “hot” tumors of MSI-H, and related research is ongoing (12). For example, combining PD-1/PD-L1 antibodies with regorafenib: in the REGOMUNE study, combining regorafenib with avelumab for the treatment of MSS CRC patients demonstrated that this combination therapy can enhance anti-tumor immunity; in the REGOTORI study, the combination of regorafenib with toripalimab for the treatment of MSS mCRC showed efficacy but did not reach statistical significance. Radiation therapy can improve the tumor microenvironment by activating certain tumor-associated immune cells, thereby synergistically enhancing anti-tumor effects. This may increase the responsiveness of locally advanced CRC to immunotherapy (39, 40). The VOLTAGE-A study aimed to explore the value of combining radiochemotherapy with immunotherapy in MSS CRC. The study found that sequential treatment with radiochemotherapy followed by the PD-1 antibody nivolumab resulted in 11 out of 37 MSS patients achieving a pathological complete response (pCR), accounting for 30% of the cases (41). Additionally, the combination of anti-EGFR treatment with immunotherapy is under investigation. According to the AVETUX trial, the combination of mFOLFOX6 with cetuximab and atezolizumab, used in patients with RAS/BRAF wild-type mCRC, yielded a higher response rate in patients with MSS status. However, responses mainly occurred within the first eight weeks and require further evaluation in randomized trials (42). Furthermore, new treatment modalities involving therapeutic combinations with tumor vaccines, CAR-T cells, etc., aimed at promoting immune activation and T cell priming are promising clinical strategies (43).

The primary limitation of our analysis is the small number of included studies, which could limit the robustness and generalizability of our findings. After a rigorous selection process, only four studies were ultimately included from an initial pool of over 6,000 reports. Such a drastic reduction may introduce selection bias, as only studies meeting strict predefined inclusion and exclusion criteria were considered. Moreover, the relatively narrow scope of the included studies (specifically limited to patients with MSI-L/MSS/pMMR status) restricts the applicability of our findings to broader patient populations, particularly those with differing molecular characteristics such as MSI-H or dMMR subgroups.

Additionally, given the small sample sizes and the limited number of trials included, caution is warranted in interpreting the results. Small trials typically possess limited statistical power and may not fully reflect the true magnitude and variability of treatment effects. Although our pooled analysis indicated low statistical heterogeneity, variability in study design, follow-up duration, outcome assessment methods, and potential biases in reporting may still contribute to unaccounted heterogeneity and influence the observed clinical outcomes.

Importantly, the diversity of biological features within MSI-L/MSS/pMMR tumors is an additional source of clinical variance and represents an inherent confounding factor. Differences in TMB, MHC expression, and immune-related gene signatures have been suggested as critical contributors to variability in patient responses to chemoimmunotherapy. Variations in TMB levels can significantly affect neoantigen production and tumor immunogenicity, potentially predicting divergent therapeutic responses (44).Similarly, differing patterns of MHC class I expression could substantially influence tumor antigen presentation, T-cell engagement, and subsequent immunotherapy effectiveness (45, 46).Immune-related gene signatures capturing activated immune signaling pathways (e.g., interferon-gamma signaling and T-cell-inflamed signatures) may further delineate subgroups with a higher likelihood of favorable clinical outcomes (47).Unfortunately, our analysis was constrained by too few studies to allow robust subgroup or sensitivity analysis, limiting the ability to explore and elucidate these potentially crucial biomarkers fully.

Collectively, these limitations underscore the critical need for further investigation. We explicitly acknowledge these constraints in our manuscript and strongly recommend future clinical trials designed with larger and more representative cohorts. Such studies can more comprehensively address these confounding factors by incorporating thorough assessments of molecular biomarkers—including TMB, MHC expression patterns, and immune-related transcriptomic signatures—to better define factors predicting favorable therapeutic responses among MSI-L/MSS/pMMR patients. Additionally, longer follow-up periods in future trials are needed to establish more definitive evidence regarding overall survival benefits and long-term safety profiles, particularly concerning potential delayed adverse effects or late-onset immune-mediated toxicities.

## 5 Conclusion

In summary, chemoimmunotherapy demonstrated certain advantages over chemotherapy in treating MSI-L/MSS/pMMR type mCRC. However, this regimen was associated with a slight increase in both all-grade and high-grade toxicity. Chemoimmunotherapy has the potential to become an effective treatment for MSI-L/MSS/pMMR mCRC. Future research should prioritize the identification of predictive biomarkers and explore novel treatment strategies to enhance outcomes in this challenging patient population.

## Data Availability

The original contributions presented in the study are included in the article/supplementary material. Further inquiries can be directed to the corresponding authors.
